# Hepatocyte Growth Factor/c-Met Signaling in Head and Neck Cancer and Implications for Treatment

**DOI:** 10.3390/cancers9040039

**Published:** 2017-04-24

**Authors:** Natalie J. Rothenberger, Laura P. Stabile

**Affiliations:** 1 Department of Pharmacology & Chemical Biology, University of Pittsburgh, Pittsburgh, PA 15213, USA; njr31@pitt.edu; 2 University of Pittsburgh Cancer Center, Pittsburgh, PA 15213, USA

**Keywords:** head and neck squamous cell carcinoma, HGF, c-Met, EMT, HPV, targeted therapies

## Abstract

Aberrant signaling of the hepatocyte growth factor (HGF)/c-Met pathway has been identified as a promoter of tumorigenesis in several tumor types including head and neck squamous cell carcinoma (HNSCC). Despite a relatively low c-Met mutation frequency, overexpression of HGF and its receptor c-Met has been observed in more than 80% of HNSCC tumors, with preclinical and clinical studies linking overexpression with cellular proliferation, invasion, migration, and poor prognosis. c-Met is activated by HGF through a paracrine mechanism to promote cellular morphogenesis enabling cells to acquire mesenchymal phenotypes in part through the epithelial-mesenchymal transition, contributing to metastasis. The HGF/c-Met pathway may also act as a resistance mechanism against epidermal growth factor receptor (EGFR) inhibition in advanced HNSCC. Furthermore, with the identification of a biologically distinct subset of HNSCC tumors acquired from human papillomavirus (HPV) infection that generally portends a good prognosis, high expression of HGF or c-Met in HPV-negative tumors has been associated with worse prognosis. Dysregulated HGF/c-Met signaling results in an aggressive HNSCC phenotype which has led to clinical investigations for targeted inhibition of this pathway. In this review, HGF/c-Met signaling, pathway alterations, associations with clinical outcomes, and preclinical and clinical therapeutic strategies for targeting HGF/c-Met signaling in HNSCC are discussed.

## 1. Introduction

Carcinomas of the head and neck account for an increasing number of cancer cases worldwide with an incidence of more than 500,000 new cases and 380,000 deaths annually [1]. Head and neck cancers (HNC) are classified as epithelial neoplasms of the pharynx, larynx, oral cavity, nasal cavity, and paranasal sinuses [2]. Albeit a heterogeneous disease both in regards to tumor location and genetic aberrations, histologically 90% of HNC are squamous cell carcinomas (HNSCC). Identified risk factors for the development of HNSCC are excessive alcohol consumption, tobacco use, high-risk human papilloma virus (HPV) infection and the autosomal genetic disease Fanconi Anemia [3,4,5]. Despite declining trends in smoking prevalence among Americans and increased HPV vaccination efforts among young adults, over 63,000 new cases of HNSCC are estimated to arise in the U.S. alone within the next year [6,7].

The current FDA-approved agents for the treatment of HNSCC include methotrexate, 5-fluorouracil, bleomycin, cisplatin, cetuximab, docetaxel, and most recently pembrolizumab and nivolumab. Unfortunately, despite the multitude of therapeutic agents and treatment modalities including surgical resection and radiation, the five-year survival rate for HNSCC has failed to improve over the past few decades with dismal response rates in recurrent/metastatic (R/M) patients [8,9]. Among the approved targeted therapies, cetuximab, a monoclonal antibody (MAb) to the epidermal growth factor receptor (EGFR), was the first to receive FDA approval for HNSCC patients with R/M HNSCC. EGFR was identified as an attractive molecular target for this disease with EGFR protein overexpression in more than 90% of HNSCCs and clinical observations correlating increased expression with increased risk of metastasis and poorer prognosis [10,11,12]. However, despite a moderate survival advantage when combined with a platinum-based agent or radiation therapy [13,14], cetuximab remains clinically limited in HNSCC due to intrinsic and acquired mechanisms of resistance to the EGFR blockade [15]. In addition to EGFR, upregulation of the programmed cell death ligand 1 (PD-L1) in 46–100% of HNSCC solid tumors initiated investigation of the immune checkpoint protein as another viable therapeutic target in HNSCC [16]. Pembrolizumab and nivolumab are MAbs targeting the programmed cell death receptor (PD-1), selectively blocking tumor-induced immunosuppression caused by PD-L1 activation of PD-1 [17]. Pembrolizumab and nivolumab received FDA approval in 2016 as first-line therapies for R/M HNSCC patients following clinical data indicating both agents provide sustained antitumor activity with 18% and 13% overall response rates (ORR), respectively, in addition to slight survival advantages when compared with standard of care therapies [18,19,20]. While the therapeutic benefit of these targeted and systemic treatments is evident, there remains a need to develop more efficacious therapies targeting additional oncogenic pathways promoting HNSCC pathogenesis. One pathway that has emerged as a potential target is the hepatocyte growth factor (HGF)/c-Met pathway.

The discovery of overexpression of HGF and its receptor c-Met have been reported in a majority of HNSCC tumors, precipitating multiple investigations into the tumorigenic effects of this pathway in HNSCC [21]. Activation of this pathway promotes epithelial-mesenchymal transition (EMT), a process characterized by the morphogenesis of epithelial tumor cells to acquire an aggressive phenotype enhancing cellular migration, invasion, proliferation, and metastasis in HNSCC [22]. Clinically, aberrant HGF/c-Met signaling has been associated with poor prognosis, lymph node metastasis, EGFR resistance, and potentially HPV status [23,24,25,26]. With ample evidence for the role of the HGF/c-Met pathway promoting disease progression, it has been investigated as a novel therapeutic target in HNSCC patients. Herein, we outline the biological mechanisms and carcinogenic implications of HGF/c-Met signaling in HNSCC, the effects of aberrant HGF/c-Met signaling on patient outcomes, and preclinical and clinical investigations surrounding targeted inhibition of this pathway in HNSCC.

## 2. HGF/c-Met Signaling in HNSCC

HGF is a pleiotropic plasminogen-like cytokine cytogenically located at 7q21.11 and originally discovered for its mitogenic effects on parenchymal hepatocytes [27]. Identical to the independently discovered scatter factor (SF) protein, HGF also stimulates morphogenesis and motogenesis of epithelial cells in various organs, indicating that HGF plays a critical role in tissue regeneration following injury and normal tissue development during embryogenesis [28,29,30,31]. Upon dysregulation of HGF signaling, however, the same properties that promote normal pathophysiology and tissue repair in turn promote tumorigenesis, proliferation, invasion, and evasion of apoptosis in several malignancies including HNSCC [32].

**Figure 1:**
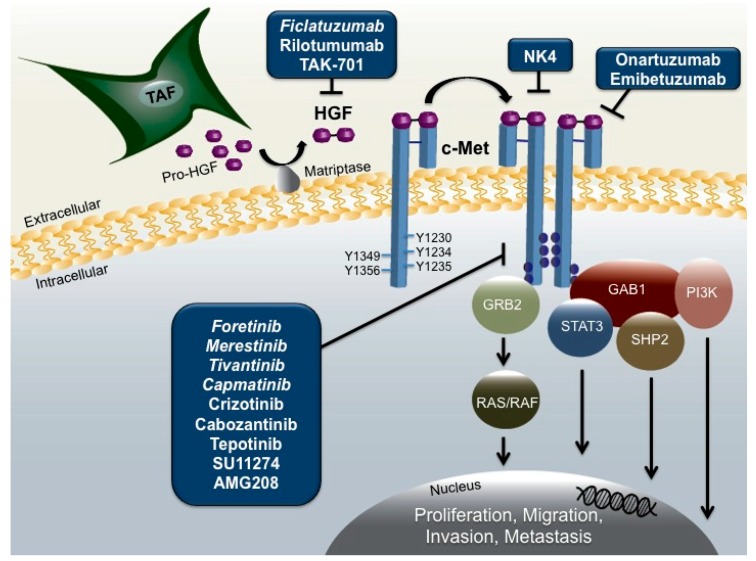
Targeted Inhibition of Hepatocyte Growth Factor (HGF)/c-Met Signaling in Head and Neck Squamous Cell Carcinoma (HNSCC). Secreted by local tumor associated fibroblasts (TAFs), pro-HGF is cleaved by membrane bound matriptase, enabling the heterodimer ligand to bind both the alpha and beta chain of the c-Met receptor. Upon binding, c-Met undergoes autophosphorylation and recruits adaptor molecules growth-factor-receptor-bound protein 2 (Grb2) and Grb2-associated binder 1 (Gab1) which further recruit oncogenic proteins SH2 containing protein tyrosine phosphatase (SHP2), signal transducers and activators of transcription-3 (STAT3), Ras/Raf, and phosphoinositide 3-kinase (PI3K), initiating signaling cascades promoting proliferation, migration, invasion, and metastasis. Inhibition of the pathway can be achieved through several strategies including anti-HGF monoclonal antibodies such as ficlatuzumab, the competitive HGF antagonist NK4, c-Met specific antibodies including onartuzumab, and c-Met tyrosine kinase inhibitors (TKIs) such as crizotinib. Italicized agents are in clinical development for HNSCC.

In HNSCC, the majority of HGF is secreted by tumor-associated fibroblasts (TAFs) in the tumor microenvironment as an inactive zymogen, requiring proteolytic cleavage by the membrane-bound protease matriptase (Figure 1) [33]. Cleavage of the single-chain proenzyme produces an active two-chain heterodimer capable of binding to the transmembrane receptor, c-Met. While autocrine HGF activation of c-Met has been reported in other cancer types, HNSCC cells fail to secrete detectable levels of the ligand, indicating the TAF supplied HGF activates c-Met in a predominantly paracrine manner [23,34].

Identified as the sole receptor for the HGF ligand, c-Met is a receptor tyrosine kinase encoded by the proto-oncogene *MET* located on the long arm of chromosome 7 at position 7q31.2 [35]. The c-Met receptor is composed of an extracellular alpha chain with a disfulfide linkage to the larger beta chain that includes a semaphorin (Sema), juxtamembrane, and cytoplasmic kinase domain integral for signal transduction [32,36]. HGF binding to c-Met leads to receptor dimerization and autophosphorylation of tyrosine residues Y1230, Y1234, and Y1235 in the active site of the tyrosine kinase domain [37,38]. Subsequent phosphorylation of tyrosines Y1349 and Y1356 located at the C-terminal of the beta chain creates a bidentate docking site that recruits and binds to the adaptor molecules, growth-factor-receptor-bound protein 2 (Grb2), and Grb2-associated binder 1 (Gab1) that are essential for downstream HGF/c-Met signaling [32,39]. Phosphorylated activation of Grb2 activates oncogenic Ras/Raf signaling, while phosphorylated Gab1 recruits docking proteins phosphoinositide 3-kinase (PI3K), SH2 containing protein tyrosine phosphatase (SHP2), and signal transducers and activators of transcription-3 (STAT-3) that activate pathways promoting cell survival, proliferation, and tumorigenesis [32].

## 3. HGF/c-Met Pathway Alterations in HNSCC

Increased activation of the HGF/c-Met signaling pathway results from a variety of genetic abnormalities including *MET* mutations, amplification of the *MET* gene, and overexpression of both c-Met and HGF protein. Overexpression of c-Met protein is the most frequently observed alteration presenting in up to 90% of HNSCC tumors, with mRNA overexpression frequently reported as well [23,40,41,42]. Activated, or phosphorylated c-Met (p-Met), is also often detected in HNSCC patient samples. In a study comparing protein expression profiles between HNSCC tumors and normal mucosa, p-Met at activating tyrosines Y1003, Y1230, Y1234, and Y1235 was observed in 66% of tumors, correlating with total c-Met expression in 79% of tumors [41]. A more recent study also reported elevated p-Met expression in 30% of HNSCC tumors and found p-Met significantly correlated with HGF protein overexpression, indicating paracrine constitutive activation of c-Met signaling by HGF in these HNSCC samples [40]. While *MET* amplification and increased *MET* gene copy number are observed at a low frequency in HNSCC tumors, they are associated with the overexpression of c-Met protein [43].

In addition to c-Met and p-Met overexpression, *MET* mutations have been identified in the *MET* tyrosine kinase domain, sema, and juxtamembrane domains in HNSCC patient tumors. In a study by Di Renzo et al., the activating point mutation Y1235D was detected at a higher incidence in metastatic lymph tissues from HNSCC patients compared to the corresponding primary tissue suggesting clonal selection of the mutation and evidence that c-Met modulates metastasis [44]. In support of these findings, a prospective study of advanced HNSCC patients reported Y1235D in 21 of 152 (14%) primary tumors with positive expression correlating to an increased likelihood of distant metastasis [45,46]. In addition, a retrospective study by Aebersold et al. observed the Y1235D mutation in 15 of 138 (11%) primary oropharyngeal squamous cell carcinomas and found tumors harboring this mutation correlated with a higher risk of local tumor progression and recurrence [46]. Subsequently, in a cohort of 66 HNSCC tumors, Seiwert et al. reported a 12% *MET* mutation frequency and identified two novel mutations (T1275I and V1333I) in the tyrosine kinase domain as well as mutations in the sema (T230M, E168D, N345S) and juxtamembrane (T1010I and R988C) domains [41]. Lastly, *MET* mutations that result in exon 14 skipping, deletion of the juxtamembrane domain, and loss of Casitas B-lineage lymphoma (Cbl) E3 ubiquitin-ligase recruitment, were only identified in less than 1% of HNSCC tumors, but potentially require more advanced technical methods for increased detection [47,48].

Paralleling c-Met expression, HGF protein overexpression has been observed in 45% of primary HNSCC tumors as well, while positive *HGF* gene expression has been reported in up to 58% of R/M HNSCC cases [40,41]. Marshall et al. were among the first to report that HGF protein expression is significantly higher in tumor tissue compared to normal and dysplastic tissue of the oral cavity [23,49]. In HNSCC, matriptase, the protease required to cleave the pro-HGF form, is also co-expressed with c-Met on the surface of HNSCC tumor epithelial cells enabling enhanced HGF-mediated activation of c-Met and downstream signaling [33]. Dysregulation of HGF/c-Met signaling in turn activates multiple effector molecules such as mitogen-activated protein kinase (MAPK) and PI3K which promote malignant growth and cell survival [23].

## 4. HGF/c-Met Pathway and HNSCC Progression

Aberrant HGF/c-Met signaling in HNSCC promotes tumor progression and enables the development of distant metastasis by increasing the invasive capacity of HNSCC tumor cells. Multiple studies have demonstrated that recombinant HGF stimulation of both primary and metastatic HNSCC cell lines leads to increased migration and invasion [23,50,51]. Similarly, HNSCC tumor cells co-cultured with TAFs, or cultured with TAF-conditioned media, also demonstrated enhanced migration and invasion, while the addition of an HGF neutralizing antibody inhibited the acquisition of a motile phenotype [23,52]. Furthermore, in vivo orthotopic injection of HNSCC cells mixed with TAFs led to increased tumor growth and metastasis compared to injection of tumor cells alone [52].

HGF/c-Met signaling stimulates the morphogenesis of epithelial cells to acquire these aggressive motile phenotypes through EMT. During EMT, cells lose their characteristic epithelial phenotype and in turn acquire mesenchymal features including front-rear polarization, an elongated spindle-like morphology, and migratory potential resulting in the metastatic progression of several carcinomas including HNSCC [53,54]. Studies assessing cell anchorage and invasion have revealed that the loss of an immobile epithelial phenotype is related to loss of E-cadherin expression [55]. A recent meta-analysis of over 19 studies assessing E-cadherin expression in HNSCC found that reduced E-cadherin expression is significantly associated with higher incidence of metastasis and poorer prognosis when compared with high E-cadherin expressing tumors [56]. Kim et al. showed HGF activation of c-Met mediated reduced membranous E-cadherin expression in HNSCC cells by causing translocation of the protein to the cytoplasm and reduced expression correlated with distant metastasis and recurrent disease [25]. Suppression of E-cadherin protein expression during tumor progression is commonly observed in response to upregulation of repressive transcription factors such as snail [57]. In a study by Grotegut et al., HGF was shown to stimulate snail expression through c-Met activation of MAPK signaling and early growth response factor 1 (EGR-1) in epithelial cells, inducing a migratory phenotype [58]. Furthermore, co-localized expression of c-Met and E-cadherin have been reported at cell-cell junctions in both breast and colon cancers [59]. Taken together, these studies suggest HGF/c-Met signaling enables epithelial HNSCC cell migration through the suppression of E-cadherin expression.

The HGF/c-Met pathway employs multiple mechanisms to induce cell migration and invasion, and in addition to E-cadherin loss, HGF induces increased expression of matrix metalloproteinases (MMPs) in HNSCC. MMPs are a class of proteases identified in tumor cell metastases and are responsible for the degradation and remodeling of the extracellular matrix (ECM) [60]. In primary HNSCC patient derived cell lines, exposure to HGF significantly upregulated expression of MMP-9, while metastatic cells from the same patients showed increased expression of MMP-2 in response to HGF [51]. Interestingly, in oral squamous cell carcinomas, snail was shown to regulate MMP-2 and MMP-9 expression, indicating HGF-induced snail expression potentially mediates simultaneous degradation of the ECM and intracellular adhesion of HNSCC cells [61,62].

Another mechanism by which HGF/c-Met signaling promotes tumor progression and cell survival is through the p53 protein product *TP53-*induced glycolysis and apoptosis regulator (TIGAR). Targeted inhibition of c-Met was found to significantly downregulate expression of TIGAR in nasopharyngeal cells in turn reducing intracellular nicotinamide adenine dinucleotide phosphate (NADPH) levels necessary for evading apoptosis [63]. Subsequent overexpression of TIGAR in HNSCC cells eliminated the growth inhibitory effects of c-Met inhibitors, while also reportedly upregulating mesenchymal markers such as vimentin [63,64]. This data indicates a relationship between c-Met and TIGAR in the promotion of cell survival and invasiveness in HNSCC.

HGF further supports HNSCC tumor development through the promotion of angiogenesis. A hallmark of cancer progression, angiogenesis is stimulated by HGF-induced production of well-established pro-angiogenic factors including interleukin (IL)-8, vascular endothelial growth factor (VEGF), and platelet-derived growth factor (PDGF). Previous reports have correlated increased IL-8 and VEGF levels in HNSCC tumors with larger tumor volume, increased recurrence and shorter disease-free intervals [65,66]. In vitro studies show that HGF stimulation leads to significantly increased IL-8, VEGF, and PDGF expression in HNSCC tumor cells through MEK-dependent activation of EGR-1 [23,67,68]. HGF-induced IL-8 and VEGF production reliant on c-Met activation of downstream MEK and PI3K pathways was further demonstrated by repressed expression of these cytokines in the presence of targeted MEK and PI3K inhibitors [23,67]. Elevated co-expression of HGF and IL-8, VEGF, and PDGF in both tumors and serum from HNSCC patients further supports the hypothesis of an interaction between HGF/c-Met signaling and these cytokines promoting angiogenesis and metastatic disease [66,67,69].

Alterations in HGF/c-Met signaling in HNSCC therefore unsurprisingly correlate with increased incidence of regional and distant metastases. While distant metastasis rates remain low, one of the most significant prognostic indicators for HNSCC patients is the presence of metastatic disease in the cervical lymph nodes, correlating with a 50% decrease in survival [70,71]. Several studies have evaluated potential metastatic biomarkers and among them HGF and c-Met protein and gene expression have been identified. HGF protein levels not only increase with HNSCC progression, but also significantly correlate with lymph node metastasis [72,73]. Additionally, c-Met protein expression is frequently observed in metastatic lymph tissue occasionally at increased levels compared with the primary HNSCC site [74,75]. While the *MET* gene is typically undetectable in normal lymph tissue, *MET* expression was found in up to 40% of HNSCC lymph nodes assessed, 24% of which were histopathologically confirmed metastatic [74]. This data, combined with reports showing somatic *MET* mutations are selectively identified in metastatic lymph, and not in the primary tissue, confirm a role of the HGF/c-Met pathway in metastasis [44].

## 5. Relation of the HGF/c-Met Pathway to HNSCC Outcome

Studies comparing c-Met expression with clincopathological parameters consistently report positive and overexpressed c-Met protein in HNSCC tumor samples [73,76,77,78]. However, the value of tumoral c-Met expression as an independent prognostic indicator in HNSCC remains controversial due to inconsistent reports regarding which clinical parameters significantly correlate with c-Met expression (Table 1). An expression analysis by Galaezzi et al. was among the first studies to assess the clinical significance of c-Met in HNSCC [75]. Utilizing both Western blotting and immunohistochemistry, elevated c-Met expression was observed in HNSCC carcinomas compared with normal squamous epithelium and correlated with increased tumor size and regional lymph node metastasis [75]. A separate study analyzing 82 laryngeal tumors reported similar findings with elevated c-Met expression significantly associated with regional lymph metastasis [79]. While subsequent studies have also reported a correlation between c-Met expression and clinicopathological parameters such as tumor stage [73,80] and local and distant metastatic recurrence [80], the most commonly reported correlations are with lymph node metastasis and decreased overall survival [40,72,73,80,81]. For example, Lo Muzio et al. evaluated c-Met expression in 84 HNSCC samples and showed a significant correlation between elevated c-Met and decreased overall survival, however no significant association was found between c-Met and staging, recurrence, or sex [77]. One of the most recent retrospective studies reported an association between c-Met and p-Met with worse progression free survival (PFS) and overall survival (OS) in metastatic HNSCC patients [40]. In contrast to these reports, an assessment of 211 HNSCC tumors failed to find any prognostic significance of c-Met expression in regards to tumor size or metastasis, and contrary to previous reports they observed a negative correlation between c-Met expression and tumor staging [78]. Furthermore, a retrospective study analyzing oropharngeal squamous cell carcinoma samples revealed a lack of statistical significance between *MET* mutations or c-Met expression and clinicopathological outcomes [82]. Despite variability among these findings, the majority of studies indicate at least one correlation between elevated c-Met and a clinical parameter indicative of poor prognosis.

HGF, both tumoral and peripheral, has also been evaluated as a potential biomarker for the progression of HNSCC (Table 2). In addition to c-Met expression, Kim et al. evaluated tumoral HGF expression and observed a significant correlation between positive HGF staining and lymph node metastasis and tumor staging [72]. Kim et al. also assessed HGF levels in the serum among healthy controls, primary HNSCC patients, and R/M HNSCC patients. This study reported a nearly 40% increase in the HGF serum levels of R/M HNSCC patients compared to healthy controls and 23% increase in nonrecurrent patients compared with controls [84]. In a study identifying therapeutic response and survival biomarkers, longitudinal changes in HGF serum levels following chemoradiation in patients with advanced HNSCC were found to significantly correlate with increased relative risk of death upon adjustment for smoking status [85]. Furthermore, a study by Uchida et al. comparing HGF serum levels in healthy volunteers to HNSCC patients, also observed significantly higher circulating HGF in patients compared to controls, however no correlation with clinicopathological parameters such as tumor size, lymph nodal status, metastasis, or prognosis was found [86]. Finally, some studies have reported trends towards improved survival rates in HNSCC patients with combined lower tumoral HGF and c-Met expression [23].

Potential explanations for the variability in these results may stem from the heterogeneity and diverse tumor sites of the HNSCCs evaluated. Furthermore, analytical parameters and cut-offs for high versus low expression levels, as well as sample size, were not consistent across all studies. Finally, confounding clinical variables, such as HPV status, were not always included in the statistical analysis. HPV is an increasingly prevalent risk factor for the development of HNSCC, with the rise in oropharyngeal cancers in the U.S. in part attributed to increased incidence of HPV infections [87]. Interestingly, patients with HPV-positive oropharyngeal tumors respond better to treatment and have longer survival rates and better OS compared with HPV-negative tumors [88]. HPV status may influence the prognostic value of c-Met expression as reported by Baschnagel et al. [76] in a study assessing 223 locally advanced HNSCC cases. A univariate analysis comparing tumors based on low vs. high c-Met expression demonstrated a significant correlation between high c-Met and decreased OS, disease free survival (DFS), and development of distant metastasis [76]. In this cohort, high c-Met was found in 73% of HPV-negative tumors but in only 27% of HPV-positive tumors. A multivariate analysis revealed this predictive correlation was only observed in patients who were also HPV-negative [76]. A separate study reported HGF overexpression in HPV-negative tumors significantly correlated with decreased OS, while HPV-positive tumors with low HGF expression associated with a more promising prognosis [26]. In this study, minimal *MET* gene amplification was found (3%), and regardless of HPV status a statistically significant correlation between prognosis and either *MET* amplification or c-Met protein expression was not observed [26]. In contrast, a recent study stratifying orpharyngeal tumors based on HPV status reported c-Met expression in 70% of samples, but found overexpression correlated with HPV-positive status, postulating the HPV-16 E6 oncoprotein upregulates c-Met protein expression in HNSCC [83]. Despite discrepancies between these clinical observations, further investigation of an interaction between HPV and the HGF/c-Met pathway in HNSCC is warranted and future studies should incorporate HPV status, if known, in multivariate analyses.

## 6. Role of HGF/c-Met Signaling as a Mechanism of Resistance to EGFR-Targeted Therapies

The EGFR-directed monoclonal antibody, cetuximab, is one of three targeted agents approved to date by the FDA for the treatment of HNSCC, and improves survival when added to front line platinum [14]. Despite abundant evidence to support EGFR inhibition as a rationale for HNSCC treatment management [11], the modest clinical benefit of cetuximab has been underwhelming [89,90] and patients either show primary resistance or develop acquired resistance over the course of treatment [91]. While analyses of *EGFR* gene copy number and protein expression in patient tumors have not been found to be predictive of cetuximab response [92,93], a well-established intrinsic or acquired resistance mechanism to anti-EGFR therapy in HNSCC is the compensatory activation of alternate receptor tyrosine kinases (RTKs) including c-Met. The HGF/c-Met signaling pathway converges with the EGFR network at both the PI3K/Akt and MAPK nodes, suggesting the ability for reciprocal compensation. Activation of c-Met can overcome EGFR blockade in preclinical models of HNSCC and in HNSCC patients [40,94,95], thereby identifying the HGF/c-Met pathway as a potential node of resistance to EGFR-targeted therapies in HNSCC.

Preclinically, the EGFR ligand TGF-α stimulated activation of c-Met in HNSCC cell lines, through prolonged tyrosine phosphorylation and increased c-Met protein expression [96]. Moreover, dual inhibition of EGFR with gefitinib and c-Met with crizotinib was necessary for maximal inhibition of phosphorylation of MAPK and Akt to effectively abrogate crosstalk between these pathways [96]. Similarly, dual inhibition significantly reduced cell proliferation, invasion, and wound healing, compared to single agent inhibition of either RTK. In vivo, dual inhibition of EGFR and c-Met retarded tumor growth, decreased the proliferative index, and enhanced apoptosis compared to either single agent [96]. A separate study demonstrated that dual blockade of c-Met with SU11274 and EGFR with erlotinib in erlotinib-sensitive HNSCC cell lines decreased viability significantly more than exposure to either single agent, and these agents acted synergistically [41]. Elevated serum HGF levels have been associated with resistance to EGFR inhibitors in *KRAS* wild-type colorectal cancer [97] and lung cancer [97,98,99], but no evidence exists for this relationship in HNSCC. In cetuximab-treated HNSCC patients, c-Met and p-Met overexpression in baseline pre-cetuximab tissues were linked with worse outcomes [40]. c-Met is also an established driver of EMT, a phenotype associated with cetuximab resistance in HNSCC [100].

Together, these data suggest that HGF/c-Met pathway inhibition may overcome resistance to anti-EGFR therapy in R/M HNSCC, such as in patients with clinical cetuximab resistance. Given the cross-talk and mutual compensation between the HGF/c-Met and EGFR signaling pathways, optimal benefit may be achieved by continuing EGFR blockade concurrent with HGF/c-Met pathway inhibition, in spite of established cetuximab clinical resistance.

## 7. Targeting the HGF/c-Met Pathway in HNSCC

### 7.1. HGF/c-Met Targeted Therapies

Several agents have been developed to target both the HGF ligand and c-Met receptor including small molecule tyrosine kinase inhibitors (TKIs), MAbs, and competitive HGF antagonists and c-Met receptor decoys (Table 3). Crizotinib (PF-2341066) is a potent c-Met TKI, inhibiting activation of HGF/c-Met signaling and all downstream effector molecules [101]. Preclinical in vitro studies using HNSCC cell lines showed crizotinib inhibited c-Met phosphorylation and wound closure in a dose-dependent manner and significantly suppressed colony formation compared with controls [23,41]. Subsequent in vivo analysis of crizotinib using HNSCC cell line xenografts revealed significant inhibition of tumor proliferation, abrogation of downstream AKT signaling, and reduced blood vessel density in the tumors [23,41]. Additionally, crizotinib was investigated in combination with an EGFR TKI, and combined inhibition of the HGF/c-Met and EGFR pathways revealed enhanced antineoplastic effects compared with singular inhibition both in vitro and in vivo [96]. Further assessed in HNSCC cancer stem-like cells (csc), crizotonib blocked csc sphere formation and revealed synergistic effects in a patient-derived xenograft (PDX) model with enhanced antitumorigenic effects when in combination with docetaxel and cisplatin [102]. However, crizotinib is also a potent inhibitor of the anaplastic lymphoma kinase (ALK) fusion protein and ALK related receptor tyrosine kinase ROS1 [103,104]. Having received FDA approval in both ALK-positive and ROS1 rearrangement-positive NSCLC tumors, these targets have become the main clinical indication for this drug. ALK fusion protein and ROS1- rearrangements are rarely observed in HNSCC, which may explain the lack of clinical investigation of this drug for HNSCC.

Other c-Met TKIs that have undergone preclinical investigation in HNSCC include foretinib, tivantinib, and SU11274. Foretinib (GSK1363089) is a multikinase inhibitor predominately targeting c-Met and VEGF receptor-2 [105]. When tested singularly in HNSCC cell lines, with and without HGF, foretinib was modestly potent (IC_50_ < 0.5 μM) [106]. However, synergy was observed when combined with the EGFR inhibitor erlotinib, resulting in enhanced anti-proliferative effects and inhibition of pathways downstream of HGF/c-Met activated signaling [106]. Tivantinib (ARQ 197) was also developed as a potent inhibitor of catalytic c-Met activity with reported efficacy in vitro and in xenograft models of multiple carcinomas [107]. A recent in vitro evaluation of tivantinib in HNSCC showed potent inhibition of proliferation and enhanced caspase-dependent apoptosis [108]. However, inhibition of c-Met phosphorylation was not observed with tivantinib compared to other agents such as crizotnib in NSCLC [109]. Preclinical assessment of additional c-Met TKIs including SU11274 showed promising in vitro efficacy, inhibiting c-Met activation and cell proliferation [23,41]. However, due to poor solubility and lack of oral bioavailability in vivo, SU11274 has not been pursued for further clinical development. Several other c-Met TKIs such as tepotinib, AMG 208, and cabozantinib have undergone preclinical and clinical investigation in other cancers, but await evaluation in HNSCC [110].

Several anti–HGF antibodies have been developed for the inhibition of c-Met mediated neoplastic effects as well. Ficlatuzumab (AV-299) is an IgG1 HGF MAb shown to selectively bind and inhibit HGF activity. After successful preclinical assessment in NSCLC, and multiple phase I clinical trials establishing a safety profile, ficlatuzumab was also evaluated in HNSCC [111]. Kumar et al. showed ficlatuzumab significantly mitigated TAF-induced migration, invasion, proliferation, and c-Met phosphorylation in HNSCC cells cultured in TAF conditioned media [112]. To date, ficlatuzumab remains the only anti-HGF antibody undergoing clinical development in HNSCC, however other HGF MAbs have reported in vitro efficacy such as rilotumumab and TAK-701. Rilotumumab (AMG 102) is a HGF IgG2 MAb that binds to the β-chain of HGF and potently decreases c-Met phosphorylation, inhibiting proliferation and invasion both in vitro and in a gliobastoma xenograft mouse model [113]. TAK-701 (L2G7) is another humanized HGF directed antibody with in vitro and in vivo antitumor efficacy as a monotherapy in NSCLC and medulloblastoma carcinomas [114,115]. Preclinical data also suggests a role for TAK-701 in overcoming HGF/c-Met induced resistance to gefitinib, while having a beneficial additive inhibitory effect when combined with gefitinib in non-resistant NSCLC carcinomas [116,117]. Despite results from a phase 1 dose-escalation study indicating tolerability of the agent, clinical development of this TAK-701 appears to have stagnated.

Other targeted agents in development for selective inhibition of HGF/c-Met signaling include Onartuzumab (MetMab), Emibetuzumab (LY2875358), and NK4. Onartuzumab is a humanized monoclonal anti-c-Met antibody designed to bind to the receptor and inhibit ligand-induced activation of the HGF/c-Met pathway [118]. Proven effective at reducing c-Met phosphorylation, cell migration, and tumor proliferation in preclinical glioblastoma models, onartuzumab has been evaluated in phaseI/II trials in solid tumors and in combination with the EGFR inhibitor erlotinib in NSCLC. Emibetuzumab, is under clinical development in phase 1 trials as a bivalent c-Met antibody, with in vitro and in vivo antineoplastic efficacy in NSCLC [119]. Characterized by its ability to not only inhibit HGF binding, but also induce c-Met internalization and degradation, Emibetuzumab also shows promise in both HGF-dependent and independent c-Met activated cancer models [119]. A competitive HGF antagonist NK4 was designed as another strategy for selective inhibition of HGF/c-Met signaling. Composed of the HGF N-terminal hairpin domain and four kringle domains, the NK4 fragment works by binding to the c-Met receptor and blocking tumor proliferation, invasion, and angiogenesis in gallbladder and pancreatic preclinical models [120]. NK4 remains to be evaluated in HNSCC.

### 7.2. Preclinical Models for HGF/c-Met Targeted Therapies

Preclinical evaluations of these agents targeted against the HGF/c-Met pathway in HNSCC predominately begin with monolayer 2-D cell culture models assessing drug efficacy, cytotoxicity, and influence on cell viability. Fortunately, a multitude of diverse HNSCC cell lines have been established allowing for a more comprehensive investigation of disease pathogenesis and druggable targets [121]. However, tumor cell lines cultured for 2-D in vitro studies fail to incorporate necessary features such as solid tumor architecture and the tumor microenvironment. To address these limitations and improve in vitro analysis of HGF/c-Met signaling in HNSCC, co-culture techniques utilizing TAF conditioned media have enabled more accurate assessment of HGF and c-Met inhibitors by incorporating the main source of HGF substrate in the assessment [23,52,112]. Furthermore, 3-D multicellular tumor spheroid models are in development to allow for a more accurate representation of tumor architecture and more precise analysis of how therapies penetrate tumor tissues [122].

Xenograft models continue to provide an effective means for identifying inhibitors for further clinical development [123]. However, due to biological incompatibility between murine ligands and human receptors, xenograft models assessing HGF/c-Met inhibition are limited. With the discovery that murine HGF (mHGF) fails to activate the human c-Met receptor, Zhang et al. developed a transgenic mouse model using human HGF (hHGF) cDNA in severe combined immunocompromised (SCID) mice [124]. Ectopic expresson of hHGF in these mice allowed for a much more accurate HGF/c-Met mouse model, however, hHGF production was observed in all tissues of this model [125]. Additionally, Francone et al. utilized adenovirus encoded with the human HGF transgene (Ad-hHGF) in SCID mice to produce a similar model limiting murine production of hHGF to the liver, while Stabile et al. used cDNA to generate a lung-specific hHGF overexpressing transgenic mouse model [109,126,127]. Development of these HGF SCID models was pivotal in the advancement of in vivo investigations of HGF/c-Met signaling enabling enhanced tumor growth of HGF-dependent cell lines and more translatable investigation of the pathway in HNSCC [125,126]. Furthermore, orthotopic models involving a submucosal injection of HNSCC into the tongue or floor of the mouth of immunocompromised mice have been developed allowing for a more representative microenvironment and assessment of regional and distant metastasis [52,128]. PDXs have also become a prevalent in vivo approach in HNSCC as they preserve tumor heterogeneity along with disease-specific genomic alterations such as *TP53* and *PI3KCA* [129,130]. Furthermore, both systemic and targeted therapies tested in HNSCC PDXs have been shown to accurately correlate with clinical response rates, even allowing for assessment in cetuximab-resistant strains [130]. These techniques have enabled translational investigation and clinical development of selective HGF and c-Met inhibitors.

### 7.3. Clinical Trials in HNSCC Targeting the HGF/c-MET Pathway

Given that the HGF/c-MET pathway is involved in multiple stages of HNSCC tumor progression, it is a highly promising therapeutic target for this disease. As discussed, many types of inhibitors have been developed to abrogate activation and signaling of this pathway, and several of these HGF/c-Met targeting agents have been evaluated in completed or on-going clinical trials for HNSCC patients as summarized in Table 4.

The first report to evaluate a c-Met inhibitor in HNSCC was a phase II study which tested the efficacy, safety, and tolerability of single-agent foretinib, an ATP-competitive inhibitor of c-Met and VEGFR2, in patients with R/M HNSCC [131]. While there was evidence of modest activity with foretinib in this population, the lack of objective response to treatment prohibited the study to continuing to the second stage of the Simon 2-stage design. Although there were no partial or complete responses in this study, half of the patients (7/14) demonstrated stable disease (SD) and 6/14 patients had minor tumor shrinkage. Furthermore, two patients had prolonged disease stabilization for more than 13 months. No biomarkers of response were evaluated due to the small sample size but the results supported the continued investigation of c-Met inhibitors for HNSCC. A dose escalation and efficacy study with the c-Met TKI, merestinib, is in progress (NCT01285037) in advanced tumors including HNSCC. In an interim report, merestinib demonstrated an acceptable toxicity profile in the dose escalation study. Single agent testing is ongoing in four tumor specific cohorts as well as an additional arm investigating merestinib in combination with cetuximab in HNSCC with a planned expansion cohort with combined cetuximab treatment [132].

Preclinical studies suggest that combination approaches with HGF/c-Met inhibition may be promising. Two subsequent studies with c-Met TKIs in combination with cetuximab are ongoing (Table 4; NCT01696955 and NCT02205398). One study utilized the highly specific c-Met non-ATP-competitive TKI, tivantinib, in combination with cetuximab versus cetuximab alone. The primary objective of this randomized phase II study is to determine the response rate of the combination versus single agent cetuximab. Biomarkers have been incorporated into the study design as well. A second study is evaluating the multi-targeted kinase capmatinib (INC280) which inhibits c-Met in addition to EGFR and HER3, in combination with cetuximab. In this phase Ib dose escalation study, the primary objective is to determine the incidence of dose limiting toxicities and to determine a maximum tolerated dose of capmatinib in combination with cetuximab. Study results have not yet been reported for these two ongoing trials with c-Met inhibitors.

Studies using HGF directed monoclonal antibodies are limited. One trial using the HGF monoclonal antibody ficlatuzumab, and one in combination with cetuximab is ongoing (Table 4; NCT02277197). This study is a phase Ib study to identify the recommended phase II dose for the combination of ficlatuzumab and cetuximab in R/M HNSCC patients. Candidate biomarkers include serum Veristat classification and tumor expression of p-Met. The results of these ongoing trials will determine whether further testing of HGF/c-Met targeting agents is justified in HNSCC. Biomarker identification and patient selection strategies to predict sensitivity to these dual-targeted studies will also be important for moving these forward clinically.

## 8. Conclusions

Head and neck cancer remains a prevalent disease in need of novel and efficacious treatment strategies for patients. Integral in the development of an invasive, proliferative, and metastatic phenotype, the HGF/c-Met pathway presents as an attractive target for molecular inhibition. Several therapies selectively targeting either c-Met or its ligand HGF have revealed potent antineoplastic effects in preclinical HNSCC models, with some agents advancing to clinical development. While clinical trials for these targeted therapies are still ongoing, inhibition of this pathway along with standardized evaluation of HGF and c-Met expression profiles with clinical outcomes is warranted in order to provide certain HNSCC patients another viable treatment strategy.

## Figures and Tables

**Table 1:** Clinical Correlations with c-Met Expression in HNSCC Tumors.

Clinicopathological Correlations with Elevated c-Met Expression	HNSCC Sample Site/Size	Reference
Lymph node metastasis	Larynx (*n* = 82)	Sawatsubashi 1998 (ref. [79])
Decreased local failure-free survival	Oropharynx (*n* = 97)	Aebersold 2001 (ref. [81])
Decreased disease-free survival		
Decreased overall survival		
Higher tumor staging	Oral Cavity (*n* = 93)	Chen 2004 (ref. [73])
Lymph node metastasis		
Clinical staging		
Decreased overall survival rate	Oral Cavity (*n* = 84)	Lo Muzio 2006 (ref. [77])
Lymph node metastasis	Hypopharynx (*n* = 40)	Kim 2006 (ref. [72])
Higher tumor stage	Tongue (*n* = 99)	Endo 2006 (ref. [80])
Lymph node metastasis		
Clinical Stage		
Local recurrence		
Distant metastatic recurrence		
Lower tumor staging	Oral Cavity (*n* = 211)	Freudlsperger 2010 (ref. [78])
Worse disease-free survival in HPV-negative patients	Oropharynx (*n* = 70)	Baschnagel 2014 (ref. [76])
	Larynx (*n* = 27)	
	Hypopharynx (*n* = 7)	
	Oral Cavity (*n* = 3)	
Decreased progression-free survival * Decreased overall survival *	Oral Cavity (*n* = 7)	Madoz-Gurpide 2015 (ref. [40])
	Oropharynx (*n* = 7)	
	Hypopharynx (*n* = 6)	
	Larynx (*n* = 12)	
	Occult (*n* = 1)	
Higher tumor staging	Oropharynx (*n* = 78)	Qian 2016 (ref. [83])
HPV-positive status		

Clinical studies were identified by a literature search using the keywords c-Met, clinicopathological correlations, HNSCC. Studies presented in the table were selected based on results with a *p* value <0.05. * Also correlated with elevated p-Met tumor expression.

**Table 2:** Clinical Correlations with HGF Expression in HNSCC Tumors or Serum.

Clinicopathological Correlations with Elevated Serum/Tumoral HGF	HNSCC Sample Site/Size	Reference
Elevated serum HGF correlates with cancer burden	Oral Cavity (*n* = 31)	Uchida 2001 (ref. [86])
Elevated tumoral HGF correlates with metastasis		
Elevated tumoral HGF correlates with lymph node metastasis and pathologic stage	Hypopharynx (*n* = 40)	Kim 2006 (ref. [72])
Elevated serum HGF correlates with higher tumor staging	Oral Cavity (*n* = 22)	Kim 2007 (ref. [84])
	Larynx (*n* = 21)	
	Oropharynx (*n* = 16)	
	Hypopharynx (*n* = 14)	
	Maxilla (*n* = 5)	
Longitudinal increases of serum HGF correlate with decreased cause-specific survival	Oropharynx (*n* = 30)	Allen 2007 (ref. [85])

Clinical studies were identified by a literature search using the keywords HGF, clinicopathological correlations, HNSCC. Studies presented in the table were selected based on results with a *p* value <0.05.

**Table 3:** Drugs Targeting the HGF/c-Met Axis.

Drug	Primary Molecular Targets	Stage in Clinical Development
**c-Met TKIs**		
Crizotinib (PF 2341066)	c-Met/ALK/ROS-1	FDA approved for ALK-Positive/ROS-1 rearrangement-positive NSCLC Preclinical: HNSCC
Foretinib (GSK 1363089)	c-Met/VEGFR2	Phase II: R/M breast cancer, papillary renal-cell carcinoma, NSCLC, metastatic gastric cancer, R/M HNSCC
Tivantinib (ARQ 197)	c-Met	Phase III: hepatocellular carcinoma Phase II: HNSCC
SU11274	c-Met	Preclinical: HNSCC and NSCLC
Tepotinib (EMD 1214603)	c-Met	Phase II: NSCLC
AMG 208	c-Met/VEGF	Phase I: advanced solid tumors
Cabozantinib (XL 184)	c-Met/VEGFRs/AXL	FDA approved for medullary thyroid cancer and advanced renal cell carcinoma patients with prior angiogenic therapy
**HGF Antibodies**		
Ficlatuzumab (AV-299)	HGF	Phase II: NSCLC Phase Ib: HNSCC
Rilotumumab (AMG 102)	HGF	Phase II/III: combined with erlotinib in recurrent stage IV squamous cell lung cancer Preclinical: glioblastoma
TAK-701 (L2G7)	HGF	Phase I: advanced solid tumors Preclinical: HNSCC
**c-Met Antibodies**		
Onartuzumab (MetMab)	c-Met	Phase III: in combination with oxaliplatin in metastatic gastroesophageal cancer and in combination with erlotinib in advanced NSCLC
Emibetuzumab (LY 2875358)	c-Met	Phase II: NSCLC, advanced gastric cancer
**HGF Antagonists**		
NK4	c-Met	Preclinical: gallbladder, pancreatic, myeloma carcinomas

All targeted therapies were preclinically and clinically evaluated for selective inhibition of HGF and c-Met. Primary molecular targets as determined by nanomolar potency of each agent are included. Most advanced stage in overall clinical development and HNSCC clinical development is listed.

**Table 4:** Clinical Trials assessing HGF/c-Met Targeted Therapies in R/M HNSCC.

Clinical Trial	Phase	HGF/c-Met Agent	Other Agents	Setting/Status
**Single agent**				
NCT00725764	II	Foretinib (GSK1363089)	-	R/M/Completed [131]
NCT01285037	I	Merestinib (LY2801653)	-	R/M/Ongoing
**Dual agent**				
NCT01696955	II	Tivantinib (ARQ 197)	Cetuximab	c-Met positive; R/M/Ongoing
NCT02205398	Ib	Capmatinib (INC280)	Cetuximab	R/M/Ongoing
NCT02277197	Ib	Ficlatuzumab (AV-299)	Cetuximab	R/M/Ongoing

Ongoing or completed clinical trials evaluating HGF/c-Met targeted therapies in R/M HNSCC.
